# Behavioural Changes in a Patient With Schizoaffective Disorder

**DOI:** 10.1192/bjo.2022.372

**Published:** 2022-06-20

**Authors:** Anto Varughese

**Affiliations:** Norfolk and Suffolk NHS Foundation Trust, Norwich, United Kingdom

## Abstract

**Background:**

A 46-year-old man has a diagnosis of schizoaffective disorder complained of intermittent abdominal pain for many years. Due to this, he had been reviewed by the GP and he was prescribed medication to help with his intermittent abdominal pain.

**Case Report:**

Over the years the abdominal pain gradually worsened. He also has communication issues due to language barriers and was unsettled for most of his assessment.

His past medical history includes a duodenal ulcer, infected swollen legs and recurrent urinary tract infections.

He continued to have pyrexia despite being on regular paracetamol. Following his second episode of pyrexia, he was referred to the hospital for further investigation.

This was found to be acute acalculus cholecystitis, with possible cholecystocolic fistula and pneumonia. He was managed conservatively with intravenous antibiotics and is awaiting cholecystectomy.

**Discussion:**

Behavioural change in people with mental illness need not necessarily be linked to their mental state as it can very well be the atypical manifestation of physical illnesses- some of which could be fatal. Prompt recognition and referral to acute medical or surgical services is essential. Staff need training in bias, diagnostic overshadowing and atypical presentations in those with mental illness which will help reduce rates of avoidable morbidity and premature mortality.

In any case physical illnesses may not present typically. Acalculous cholecystitis is a rare type of gall bladder inflammation and the cause in Mr X's case is not clear. At times of COVID-19, with the anxieties around exposure to hospitals and infections, it is important to be aware of this and ensure that people with worrying physical symptoms are promptly referred whether or not it is considered to be related to COVID-19.

**Conclusion:**

Due to the pandemic, we were cautious on the ward and community about COVID-19 and preventing catching and spreading the infection. During all this, patients change of behaviour shouldn't be alluded to deterioration of mental health and mental health professionals should also consider ruling out physical causes for the change of presentation.

